# A Hamiltonian Replica Exchange Molecular Dynamics (MD) Method for the Study of Folding, Based on the Analysis of the Stabilization Determinants of Proteins

**DOI:** 10.3390/ijms140612157

**Published:** 2013-06-06

**Authors:** Massimiliano Meli, Giorgio Colombo

**Affiliations:** Istituto di Chimica del Riconoscimento Molecolare, CNR, Via Mario Bianco 9, Milano 20131, Italy; E-Mail: massimiliano.meli@icrm.cnr.it

**Keywords:** protein folding, molecular dynamics, protein stability, replica exchange

## Abstract

Herein, we present a novel Hamiltonian replica exchange protocol for classical molecular dynamics simulations of protein folding/unfolding. The scheme starts from the analysis of the energy-networks responsible for the stabilization of the folded conformation, by means of the energy-decomposition approach. In this framework, the compact energetic map of the native state is generated by a preliminary short molecular dynamics (MD) simulation of the protein in explicit solvent. This map is simplified by means of an eigenvalue decomposition. The highest components of the eigenvector associated with the lowest eigenvalue indicate which sites, named “hot spots”, are likely to be responsible for the stability and correct folding of the protein. In the Hamiltonian replica exchange protocol, we use modified force-field parameters to treat the interparticle non-bonded potentials of the hot spots within the protein and between protein and solvent atoms, leaving unperturbed those relative to all other residues, as well as solvent-solvent interactions. We show that it is possible to reversibly simulate the folding/unfolding behavior of two test proteins, namely Villin HeadPiece HP35 (35 residues) and Protein A (62 residues), using a limited number of replicas. We next discuss possible implications for the study of folding mechanisms via all atom simulations.

## 1. Introduction

The investigation of the mechanisms underpinning protein structure formation and stability represents a central interest for structural biology. Experimental and theoretical approaches have provided a large amount of information into the kinetic and thermodynamic aspects of the process. Among these, atomic resolution molecular dynamics (MD) simulations allow, in principle, the study of the protein folding reaction in full detail, shedding light on possible intermediate states and folding pathways.

However, sampling with classical MD is known to be limited to specific low energy regions of the conformational space. An additional problem is represented by the presence of multiple energy minima, separated by free-energy barriers, whose heights are often larger than the thermal energy available to the system, causing trajectories to be trapped and confined to local minima.

To overcome these limitations, several approaches to increase conformational sampling have been developed over the years: simulated annealing [1], potential scaling [2–4], locally enhanced sampling [5], parallel tempering [6], hyperdynamics [7], metadynamics [8] and the adaptive biasing force method have all been proposed to overcome sampling limitations during molecular simulations.

The parallel tempering, or the replica exchange MD (REMD) method, has emerged as one of the most widely used methods to enhance conformational sampling [9,10]. Several replicas of the system are simulated independently in parallel, under different conditions. At predefined intervals, neighboring pairs of replicas are exchanged with a specific transition probability. In most REMD studies, the temperature is used as the parameter that changes among the replicas. Exchanging temperatures allows conformations that are trapped in local minima at a low temperature to escape by passing to a higher temperature condition.

Efficient exchange between neighboring replicas requires overlap of the potential energies sampled at nearby simulation temperatures. This results in the necessity of simulating high numbers of replicas when studying protein systems in explicit solvent. Indeed, the number of required replicas grows as the square root of the number of particles in the system (to cover a desired temperature range) [11]. In addition, a larger number of replicas, in turn, requires increased simulation times to allow efficient “diffusion” of replicas in temperature space. This clearly limits the potential applications of the method, as it results in high demands in terms of computing power.

A possibility to alleviate the limitations of temperature based replica exchange has been provided by Hamiltonian REMD (H-REMD), in which the different replicas are simulated (in most cases) at a constant temperature, while the force field or Hamiltonian of the system are used as a replica-coordinate [11–14]. This approach is based on the consideration that, since the different simulations are parallel and non-interacting, it is not strictly necessary to use the same Hamiltonian for all of them.

If we imagine restricting the changes introduced in the different Hamiltonians to only a subset of the degrees of freedom of the system, the number of replicas needed to cover a given range in “effective temperature” can be greatly reduced compared to T-REMD simulations. Methodological and technical details can be found in dedicated reviews [15].

As a notable example, Zacharias has introduced the so-called Biasing Potentia-Replica Exchange (BP-REMD) method, which focuses on the protein backbone transitions as a replica coordinate. The biasing potential reduces the energy barriers associated with peptide backbone dihedral transitions. The level of biasing is gradually changed along the replicas such that frequent transitions are possible at high levels of biasing and the system can escape from local energy minima [14].

Affentranger and coworkers reported the implementation of a new H-REMD protocol based on the simultaneous modification of electrostatic and Lennard-Jones (LJ) parameters [16]. This approach is based on the consideration that the dynamic properties of a protein are influenced by its interparticle non-bonded interactions [17]. Modifying force-field parameters for such interactions should, in principle, lead to enhance the sampling of the conformational space by directly influencing the interactions of the system and, therefore, its dynamic properties.

The authors implemented a scheme that uses modified force-field parameters to treat interparticle non-bonded potentials within the protein and between protein and solvent atoms, leaving unperturbed those relative to solvent-solvent interactions. Direct comparison between the H-REMD results and classical MD results provides a clear enhancement of the sampling of rare events, such as unfolding and refolding, and the possibility of visiting high-energy conformations [16].

In this paper, we have set out to combine the concepts of H-REMD based on the modification of non-bonded parameters governing interparticle interactions, with the information on the residues identified as most important for the stabilization of a certain structure, provided by the Energy Decomposition Method [18–20]. In the latter method, we compute the non-bonded interaction energy matrix of the structure to be examined. After diagonalizing this matrix, the analysis of the principal eigenvector allows for the identifying of the essential residues and non-bonded interactions that define the folding core (see Methods). The method has been validated in several applications, which also allowed the design of new sequences with specific functions [20–25].

If the identified residues are actually fundamental for stability, perturbing their non-bonded interactions with all other residues of the protein, and the solvent particles via a modification (smoothening, soft core potential) should, in principle, destabilize the fold. Restoring the “real” interparticle interactions, on the other hand, is expected to bring the protein back into the native folded structure. The implementation of this approach in a replica exchange MD scheme has the potential to improve the exploration of the complex energy surface of proteins and to provide information on the structures of possible mis-folded, un-folded and intermediate structures.

We have used two proteins, namely the Villin HeadPiece HP35 (35 residues) and Protein A (62 residues), as the first test cases for our methodology. We report the characterization of their conformational dynamics behavior, showing the reversibility of folding-unfolding transitions at 300 K in the reference replica, corresponding to the original force field parameters.

## 2. Results and Discussion

The aim of H-REMD approaches is to enhance conformational space sampling compared to classical MD. Different types of H-REMD approaches have been proposed. The use of Hamiltonians with modified non-bonded interaction parameters introduced by Affentranger and coworkers is particularly interesting, as it enhances conformational space sampling by affecting the interactions that most directly determine the dynamic properties of the system [16,17]. In this work, we have asked the question of whether it is possible to push conformational sampling by modifying only the properties of the residues that are most important to determine the fold of a certain protein and to stabilize its structure.

The starting hypothesis is that energy is unevenly distributed in protein structures. In other words, some inter-residue interactions contribute more than others to the stabilization of the fold. Indeed, a number of experiments on many different proteins have proved that the modification of specific residues results in a dramatic change of the stability properties of the molecule, while the perturbation of other positions does not show any impact. The energy decomposition method has been introduced with the aim of identifying the residues that contribute the most in stabilizing the 3D structure of a protein and whose modification results in a stability change. The method provides a simplified view of residue-residue pair interactions, extracting the main contributions to energetic stability of the native structure from the results of all-atom MD simulations (see Materials and Methods). Analysis of the *N* (*N* corresponds to the number of residues in the protein) components of the eigenvector associated with the lowest eigenvalue, obtained after diagonalization of the full energy matrix, determines the residues behaving as strong interaction centers. This is achieved by selecting those characterized by components with an intensity higher than the threshold value corresponding to a “flat” normalized vector, whose residues would all provide the same contribution. The strong interaction centers are defined as folding hot spots.

**Figure 1:**
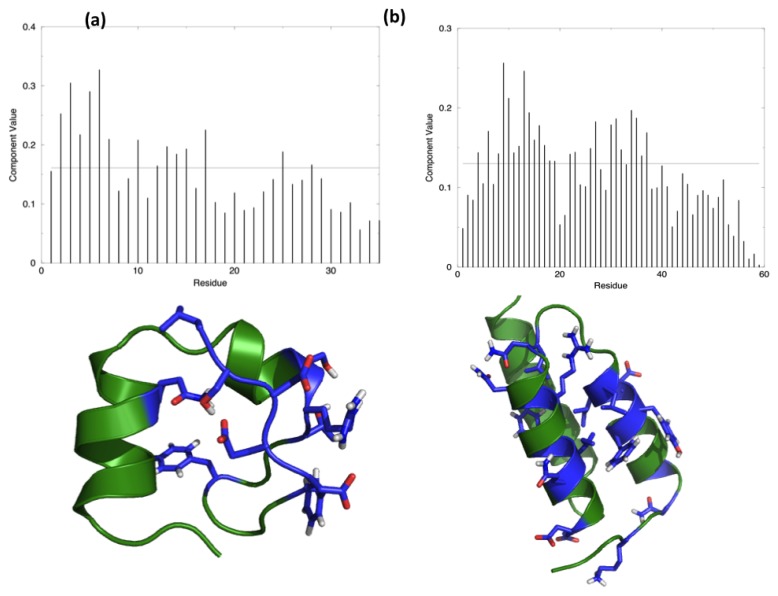
Representation of the components of the main eigenvector and projection of the identified hot spots on the 3D structure of the two simulated proteins: (**a**) HP35; (**b**) Protein A. The hot spots are evidenced in stick representation and blue color.

Figure 1 shows the distribution of the hotspots on the structures of the two test proteins, HP35 and Protein A, together with a representation of the respective lowest eigenvector.

**Figure 2:**
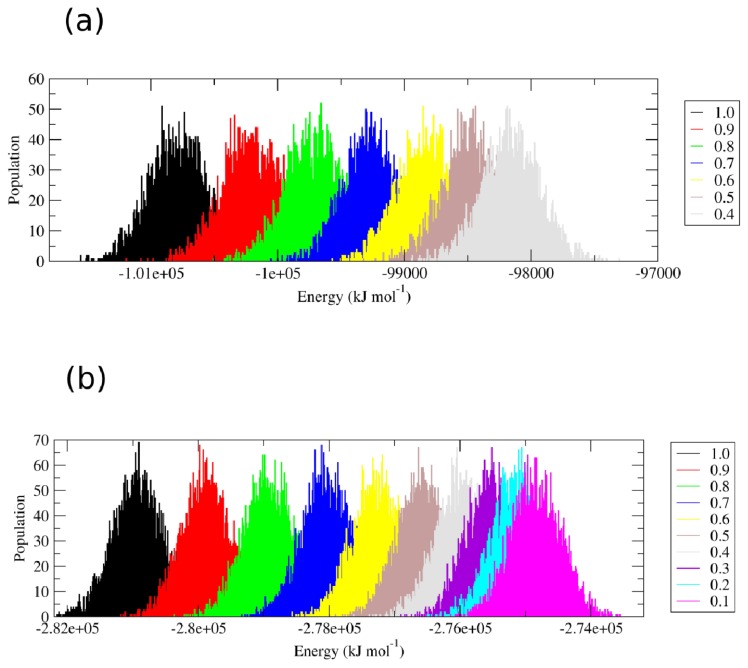
Histograms of the potential energies for the individual replicas, as obtained from the whole Hamiltonian-replica exchange molecular dynamics (H-REMD) simulations for (**a**) HP35 = and (**b**) Protein A. The left-most and right-most curves correspond to the structural ensembles simulated, respectively, with the unmodified and most strongly modified force fields. The curves for neighboring force fields overlap considerably, ensuring large replica-exchange probabilities

Perturbation of the interparticle non-bonded interactions for these residues with soft core potentials in the replica exchange scheme developed by Affentranger and coworkers [16] favors the transition of the two proteins into conformational states that are highly different from the native one. Restoring the “real” force field interactions, characteristic of the first replica, brings the protein back into the folded state. The energetic overlap between the different replicas is shown in Figure 2.

**Figure 3:**
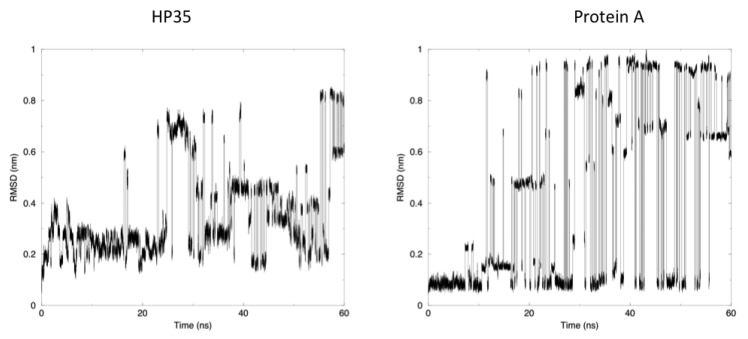
Time-dependent evolution of the RMSD of the backbone atoms from the reference crystal for each protein in the unmodified force-field replica.

The time evolution of the root mean square deviation (RMSD) from the experimental structures, calculated for replica 1, is shown in Figure 3. In both the case of HP35 and Protein A, there is an equilibrium between folded (RMSD from native lower than 0.2 nm) and unfolded conformations. In the latter case, RMSD reaches around 0.6–0.8 nm, before falling back to low values in multiple instances and in a reversible way.

Cluster analysis of the structures visited during the simulations shows that both proteins are able to visit multiple compact conformations, with the formation of different secondary structure motifs.

**Figure 4:**
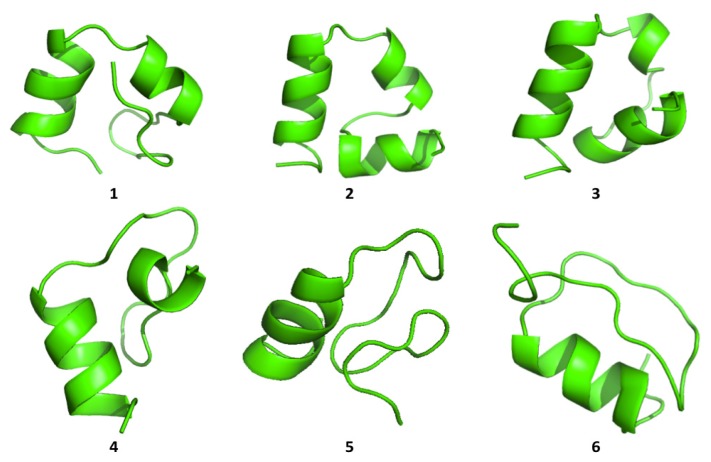
The representatives of the six most populated structural clusters for HP35.

Interestingly, HP35 populates beta-sheet rich structures, which span the *N*-terminal part of the sequence corresponding to the original helices, 1 and 2 (Figure 4). The formation of this type of non-native structure has been previously observed in experimental and computational studies of the system.

**Figure 5:**
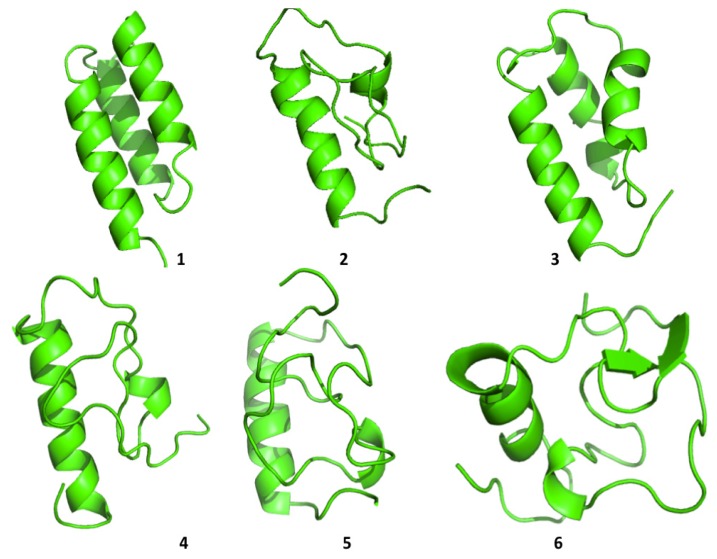
The representatives of the six most populated structural clusters for Protein A.

Protein A also shows a tendency to populate a range of different conformations (Figure 5). In general, the protein tends to refold, starting from the *C*-terminal a-helix, in agreement with some experimental data [26] and with simulation results proposed by Maisuradze and co-workers [27]. It must be underlined, however, that the folding mechanism of Protein A appears to be dependent on the conditions in which the reaction is studied, such as temperature, solvent viscosity, *etc*.

**Figure 6:**
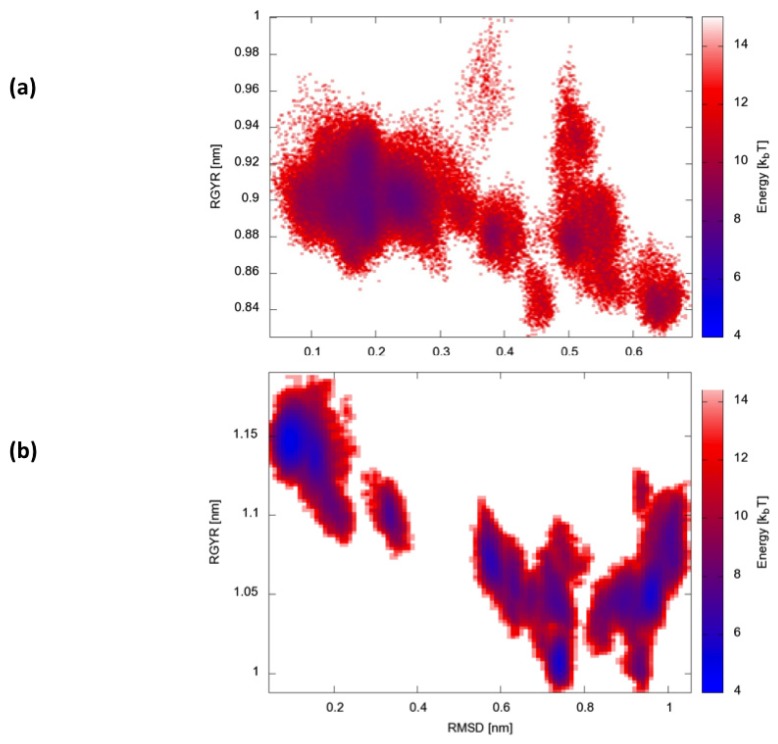
Two-dimensional representations of the free-energy landscapes for the two simulated proteins calculated on the root mean square deviation (RMSD) of the backbone atoms from the reference structure and the radius of gyration of the proteins. (**a**) HP35; and (**b**) Protein A.

Finally, we calculated two-dimensional free-energy landscapes (FEL) obtained by projecting the trajectories on the plane defined by the parameters that best describe the dynamic conformational evolution of the two proteins, namely the backbone RMSD from the native state and the radius of gyration (Rgyr) (Figure 6). Interestingly, HP35 appears to evolve towards a large basin corresponding to native-like conformations. Protein A, in contrast, visits multiple different structural ensembles, consistent with the possibility to exploit different condition-dependent mechanisms for folding.

### Mechanistic Considerations, Potentials and Limitations of the Proposed Approach

The energy decomposition-based H-REMD method presented here has proven efficient in sampling different accessible structures. In principle, this allows us to evaluate the relative weight of (clusters of) low energy conformations and to reconstruct the underlying free-energy landscape, providing valuable information on the thermodynamic aspects of folding and conformational changes. However, it must be clearly underlined that, since the potential is modified in order to favor transitions between replicas, the folding pathways and the general aspects of the folding mechanism are significantly altered. In other words, it is not possible to extract kinetic information from the simulations presented here. Indeed, the interactions that are modified correspond to a small subset of native-state stabilizing contacts. This biases the folding-unfolding mechanisms favoring the formation/disruption of native contacts and disfavoring non-native ones. As a consequence, our approach would be inefficient or unable to reconstruct folding pathways that strongly depend on the formation of non-native contacts and pass through non-native intermediates. Such limitations are common to methods that rely on information on the native state of proteins. This may not necessarily be a problem, if one is clearly aware that the goal is to explore accessible states alternative to the folded one and not to carry out structure prediction of kinetic analyses.

Another mechanism-related limitation that arises from the analysis of our simulations is that in most cases, the systems become trapped in local minima and must exchange among different replicas to escape and undergo conformational transitions. This gives rise to the sharp transitions shown in Figure 3, where alternative conformations possible with the native force field parameters and at the room temperature are identified after traveling through different replicas. It is worth noting once more that this does not represent a true folding-unfolding trajectory, but rather the switching between different structures. It should also be noted that such structures are accessible at the native temperature, and their sampling would not have been possible in the absence of our perturbation scheme. A 100 ns control simulation of HP35 run with “real” force-field parameters at 300 K visits mainly the native conformation, as expected.

Despite all these limitations and caveats, the method we have presented shows promise in terms of structure-sampling efficiency. We compared our scheme with normal temperature REMD: 39 replicas were necessary in order to obtain a switching probability of 0.20 between 300 and 450 K for HP35. On the one hand, this allows us to exchange between physical models (the force-field parameters are unchanged). On the other hand, however, using such a high number or replicas challenges the limits of our computational capabilities and cannot certainly be efficient. In structural terms, the results are comparable to the ones obtained with the energy decomposition-based H-REMD, which requires a much more limited number of replicas (between 8 and 12, in general, based on our observations).

The efficiency of the method is clearly apt to be improved: for instance, simulations and transitions among replicas could be optimized by the use of flow-optimization methods, such as the ones presented by Nadler *et al.* [28]. The method and the results presented here constitute, indeed, the starting point for the development of an enhanced sampling methodology that can be effectively and generally applied to the study and characterization of the conformational properties of polypeptides and small proteins.

## 3. Experimental Section

### 3.1. MD Simulations and Energy Decomposition

The staring structures for MD simulations are taken from the Protein Data Bank, with the following codes: 1yrf.pdb for HP35 and 1ss1.pdb for Protein A.

For each structure, after a 1,000-step minimization using the Steepest Descent algorithm, 20 ns molecular dynamics NVT simulation in a octahedral water box with explicit solvent and periodic boundary conditions are run using the GROMACS package (version 3.2.1) [29–31], with the GROMOS96 43A1 force field [32]. The simple point charge model, SPC, is used to model water molecules [33]. All bond lengths are constrained by means of the LINCS algorithm [34]. Electrostatic interactions are treated via PME implementation of the Ewald summation method. Temperature is set to 300 K and controlled by Berendsen thermostat [35]. The time step is set to 2 fs.

The energy decomposition method is based on the calculation of an interaction matrix, *M_ij_*, obtained by averaging the interaction energies between residue pairs, comprising all the non-bonded inter-residue atomic energy components (namely, van der Waals and electrostatic), over a MD trajectory starting from the native conformation. Average interactions are calculated over the equilibrated part of each MD trajectory. For the sake of homogeneity, this calculation was carried out on the last 15 ns of each simulation. For the mutants considered here, the equilibration phase is rather short and comprises only the first 2–3 ns. Running the calculation on the whole trajectory leads to the same results, given the relatively short equilibration period of the simulations and the fact that the mutations introduced do not cause major rearrangements in the three-dimensional structures of the proteins. In this calculation, diagonal elements, containing self-interactions, are neglected. The matrix, *M^ij^*, can be diagonalized and re-expressed in terms of eigenvalues and eigenvectors, in the form:

(1) $$M_{ij}=\sum_{k=1}^{N}\lambda_{k}w_{i}^{k}w_{j}^{k}$$

where *N* is the number of amino acids in the protein, λ*_k_* is an eigenvalue and *w_i_^k^* is the *i*-th component of the associated normalized eigenvector. Eigenvalues are labelled following an increasing order, so that λ_1_ is the most negative. In the following, we refer to the first eigenvector as the eigenvector corresponding to the eigenvalue λ_1_. The total non-bonded energy *E_nb_* is defined as:

(2) $$E_{nb}=\sum_{i,j=1}^{N}M_{ij}=\sum_{i,j=1}^{N}\sum_{k=1}^{N}\lambda_{k}{w_{i}}^{k}{w^{k}}_{j}=\sum_{k=1}^{N}\lambda_{k}W_{k}$$

where $W_{k}=\sum_{i,j=1}{w_{i}}^{k}{w^{k}}_{j}$. If λ_1_*W*_1_ is much larger than λ*_k_W_k_* for *k* ≠ 1, the sum over *i,j* of *M_ij_* is dominated by the contribution, due to the first eigenvalue and eigenvector, such that the total non-bonded energy can be approximated by:

(3) $$E_{nb}\approx {E^{app}}_{nb}=\lambda_{1}\sum_{i,j=1}^{N}{w_{i}}^{1}{w^{1}}_{j}=\lambda_{1}W_{1}$$

The hot spots are defined as those sites whose component is higher than a threshold value, *t*, which is calculated as the value corresponding to a normalized vector whose components provide all the same contribution for each site (flat eigenvector). This corresponds to a case in which each residue contributes with the same weight to structural stability. In this approximation, the threshold value depends only on the number, *N*, of residues in the protein and is calculated as: ${w_{i}}^{1}=\frac{1}{\sqrt{N}}$ for each *i*.

### 3.2. Replica Exchange Scheme

In standard temperature REMD, different copies of the system are simulated at different temperatures (T_0_, T_1_, …, T_N_). Replicas evolve independently, and after a certain time interval, pairs of neighboring simulations are attempted, according to the Metropolis Criterion, the exchange probabilities, *w*, being:

(4) $$\begin{array}{l} w(x_{i}\to x_{j})=\text{min}[1,\text{exp}(-\Delta )]\\ \Delta =(\beta_{i}-\beta_{j})[E(r_{j})-E(r_{i})] \end{array}$$

with β = *1/RT* and *E*(*r*) representing the potential Energy of the system.

In Hamiltonian exchange REMD, condition (1) can be rewritten as:

(5) $$\begin{array}{l} w(x_{i}\to x_{j})=\text{min}[1,\text{exp}(-\Delta )]\\ \Delta =(\beta )\left[\left(E^{i}(r_{j})-E^{i}(r_{i}))-(E^{j}(r_{j})-E^{j}(r_{i})\right)\right] \end{array}$$

in which the Metropolis criterion involves only one temperature and the energy difference between neighboring configurations using the force field for replica *j* (*Ej*) minus the same difference using the force field for replica i (*Ei*).

Following Affentranger *et al.* On the basis of the GROMOS 43a1 force-field and combination rules, we generated modified force fields multiplying the charges and the C6^1/2^ and C12^1/2^ LJ parameters of the atoms of selected protein residues by a factor, *f* < 1. Different values of the factor, *f*, are employed for each replica: the spacing between factors, *f*, was chosen such that they would decrease roughly exponentially, and their exact values were tuned, by means of few short trial simulations, to yield exchange probabilities of roughly 20%.

The rescaling was applied only to the residues whose component was higher than ${w_{i}}^{1}=\frac{1}{\sqrt{N}}$ in the main eigenvector calculated with energy decomposition.

Each replica is 60 ns long.

For HP35, we used 10 replicas. For Protein A, we used 12 replicas. All analyses were carried out on the replica corresponding to the “real”, unscaled force field.

## 4. Conclusions

In conclusion, by combining the physico-chemical information on the main determinants of protein folding and stability defined obtainable from the energy decomposition method, to the enhanced sampling capabilities of replica exchange MD, the simple approach presented allows a wide exploration of the conformational space of the proteins analyzed. In particular, our Hamiltonian REMD scheme is based on the perturbation of the physical interactions and degrees of freedom that are most important for folding. This permits an efficient exploration of different conformations, while using a limited number of replicas.

We think that the scheme we have preliminarily presented here could be readily extended to the study of the folding processes of other proteins, but also to different problems in which strong interactions among limited numbers of specific amino acids are important, such as large conformational changes or protein-protein interactions.
